# Strengthening the continuum of care and multisectoral action for non-communicable diseases in Chamarajanagar district, India: an implementation research protocol (IMPACT NCD)

**DOI:** 10.12688/wellcomeopenres.24926.1

**Published:** 2025-10-29

**Authors:** Yogish Channa Basappa, Shibani Sushmitha Ray, Shwetanjali Kumari, Kaushik Siddappa, Nazia J Shekhaji, Prashanth N Srinivas

**Affiliations:** 1 Centre for Health Systems, Institute of Public Health Bengaluru, Bengaluru, Karnataka, India; 2 Family Medicine and Population Health (FAMPOP), Faculty of Medicine and Health Sciences, University of Antwerp, Antwerp, Belgium; 3 Centre of Adivasi Health, Institute of Public Health Bengaluru, Bengaluru, Karnataka, India; 4 CSR Head, Siemens Healthineers India, Mumbai, India; 5 Health and Family Welfare Services Department, Government of Karnataka, Bengaluru, Karnataka, India

**Keywords:** non-communicable diseases, behavioural risk factors, continuum of care, implementation research, health system, multisectoral action, health system strengthening, community health

## Abstract

**Background:**

Non-communicable diseases (NCDs), particularly hypertension and diabetes, represent a rising public health burden in India, driven by lifestyle changes and complex social determinants. In Karnataka’s Chamarajanagar district, previous research identified a high NCD prevalence alongside systemic gaps in the continuum of care and multisectoral action. The IMPACT NCD project is an implementation research study that aims to strengthen the primary healthcare system's capacity and foster multisectoral collaboration for effective NCD prevention and management. Its primary scientific objective is to conduct a rigorous implementation evaluation. This evaluation examines the causal pathways and contextual factors that influence the success of these real-world interventions to understand what works, for whom, and why.

**Methodology:**

The study is guided by a theory-driven implementation research design. Interventions, developed using a participatory approach, include two main components: Capacity building and technical support for healthcare workers at Ayushman Arogya Mandirs (AAMs) on NCD screening, follow-up, and the continuum of care. Sensitisation workshops for local elected officials, police personnel, and students in grades 8 to 12. The effectiveness of these strategies will be evaluated using a sequential mixed-methods approach to understand the pathways and mechanisms of change. First, quantitative program data will be analysed using Principal Component Analysis (PCA) and cluster analysis to stratify primary units by performance. Subsequently, a purposive sample of high-performing and low-performing units will be selected for in-depth qualitative analysis through interviews and observations to test and refine the project's Theory of Change.

**Discussion:**

The project’s strong focus on implementation evaluation is critical. By moving beyond simply measuring outcomes to understanding the underlying processes and contextual factors, the study aims to identify mechanisms that drive implementation success and sustainability mechanisms of change, the study will generate crucial insights into how complex, multisectoral NCD programs can be effectively embedded within existing government systems.

## Introduction

Non-communicable diseases (NCDs)—including cardiovascular diseases (such as hypertension, heart disease, and stroke), cancer, diabetes, and chronic respiratory diseases (such as chronic obstructive pulmonary disease and asthma)—are chronic conditions that constitute a significant global health burden, causing an estimated 74% of all deaths worldwide ^1– 3^. India is grappling with a dual burden of both communicable and non-communicable diseases ^4^. The burden of NCDs is rising in both urban and rural areas, affecting all socioeconomic groups, including tribal communities ^5^. From 1990 to 2019, mortality due to NCDs in India increased from 35.8% to 64.9%, and the associated Disability-Adjusted Life Years (DALYs) rose from 29.1% to 57.9% ^6^.

Hypertension and diabetes are two of the most significant drivers of this burden ^7^. Hypertension is a leading preventable risk factor for cardiovascular diseases (CVDs), which account for an estimated 27% of all deaths in India ^8^. Furthermore, the consequences of uncontrolled diabetes are severe, significantly increasing the risk of cardiovascular diseases, kidney failure, blindness (retinopathy), and nerve damage ^9^. This epidemiological transition places considerable strain on the nation's healthcare infrastructure ^3^.

India's post-1991 economic reforms (liberalisation, privatisation, and globalisation) have spurred rapid urbanisation, lifestyle shifts, and the increased availability of packaged foods, tobacco, and alcohol. These changes have contributed to the rising prevalence of NCDs like hypertension, diabetes, and cancer, even in rural communities ^10– 13^. These behavioural risk factors are not merely individual choices but are deeply rooted in social determinants—including social, cultural, political, economic, and environmental factors—that operate at a societal level to influence individual behaviour ^14– 17^. When adolescents are exposed to NCD behavioural risk factors at a young age, these risks can accumulate throughout their lives, leading to the early onset of chronic diseases ^2, 18^. Policies and programs influencing NCDs are formulated at multiple levels, from global to local, and decisions made outside the health sector often impact these behavioural risk factors ^19, 20^. Therefore, strategies for the primordial and primary prevention of NCDs require action beyond the health sector, demanding political will across various ministries and government agencies ^16, 21^. While much of the academic literature on this type of multisectoral action comes from high-income countries, India faces unique challenges due to its socioeconomic status, culture, and demography ^22, 23^.

India's national strategy for combating NCDs is spearheaded by the National Programme for Prevention and Control of Non-Communicable Diseases (NP-NCD). This comprehensive program, implemented nationwide, focuses on capacity building for human resources, health promotion, and the early diagnosis and management of NCDs. A central component is population-based screening, which mandates screening for all individuals aged 30 and above for common NCDs like hypertension, diabetes, and oral, breast, and cervical cancers. These services are delivered primarily through the Comprehensive Primary Health Care (CPHC) approach under the Ayushman Bharat initiative. The main platform for implementation is the network of Ayushman Arogya Mandirs (formerly Health and Wellness Centres), which handle community mobilisation, risk assessment, screening, and treatment initiation at the grassroots level. In line with this national framework, Karnataka state government has taken active measures to strengthen their network of Ayushman Arogya Mandirs to improve infrastructure and enhance the delivery of NCD services ^24^.

Data from the National Family Health Survey-5 (NFHS-5, 2019–2021) reveals that the prevalence of risk factors such as high systolic blood pressure, elevated random blood sugar (RBS), tobacco use, and alcohol consumption in the Chamarajanagar district is comparable to that in neighbouring districts like Mysuru and the state of Karnataka as a whole ^25– 29^. The IMPACT NCD project builds on findings from previous work in the Chamarajanagar district by the Institute of Public Health, Bengaluru. These earlier projects, "Improving the Capacity of Health System and Community for Screening and Management of Some Non-Communicable Diseases among Scheduled Tribes: An Implementation Research in Chamarajanagar district, Karnataka (INCARE)," ICMR national taskforce project, and "Towards Health Equity and Transformative Action on tribal health (THETA)," identified key challenges ^30, 31^. Phase 1 of INCARE highlighted a high burden of hypertension and diabetes alongside limited NCD awareness among both community health workers and the general populace. Phase 2 revealed additional systemic issues, including technical flaws in enumeration forms, inadequate patient follow-up systems, and substandard screening and diagnosis processes. These collective findings informed the health system-based approach developed for the current study ^30, 32, 33^.

**Figure 1.**
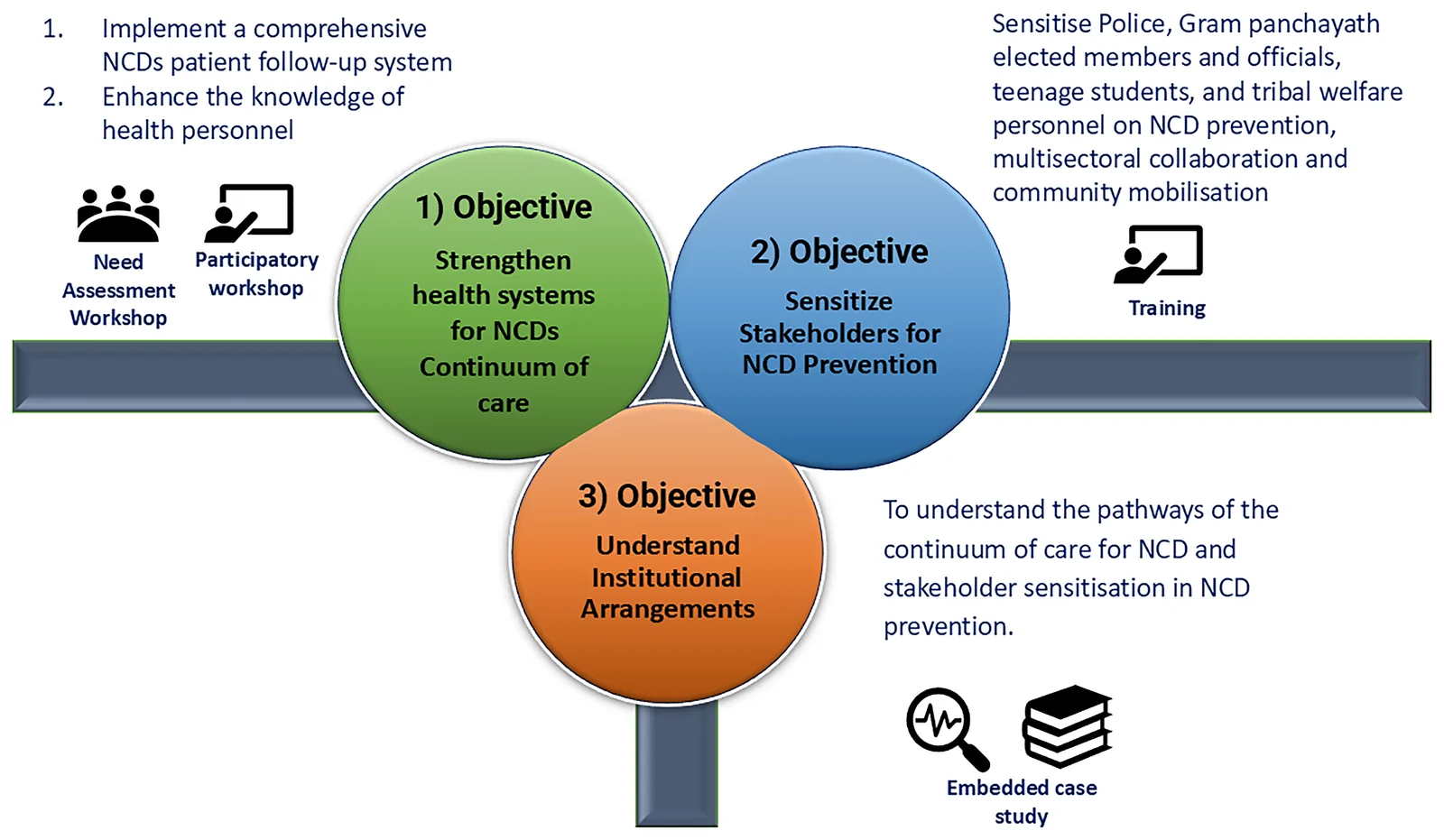
Objectives of IMPACT NCD in relation to methods.

The "Strengthening the Hypertension and Diabetes Continuum of Care and Multisectoral Action in Chamarajanagar District, India: An Implementation Research" (IMPACT NCD) project aims to enhance the capacity of healthcare systems and government agencies for the prevention and initiation of the continuum of care for hypertension and diabetes at the Ayushman Arogya Mandir. The objectives are outlined in Figure 1.

## Methods

**Figure 2.**
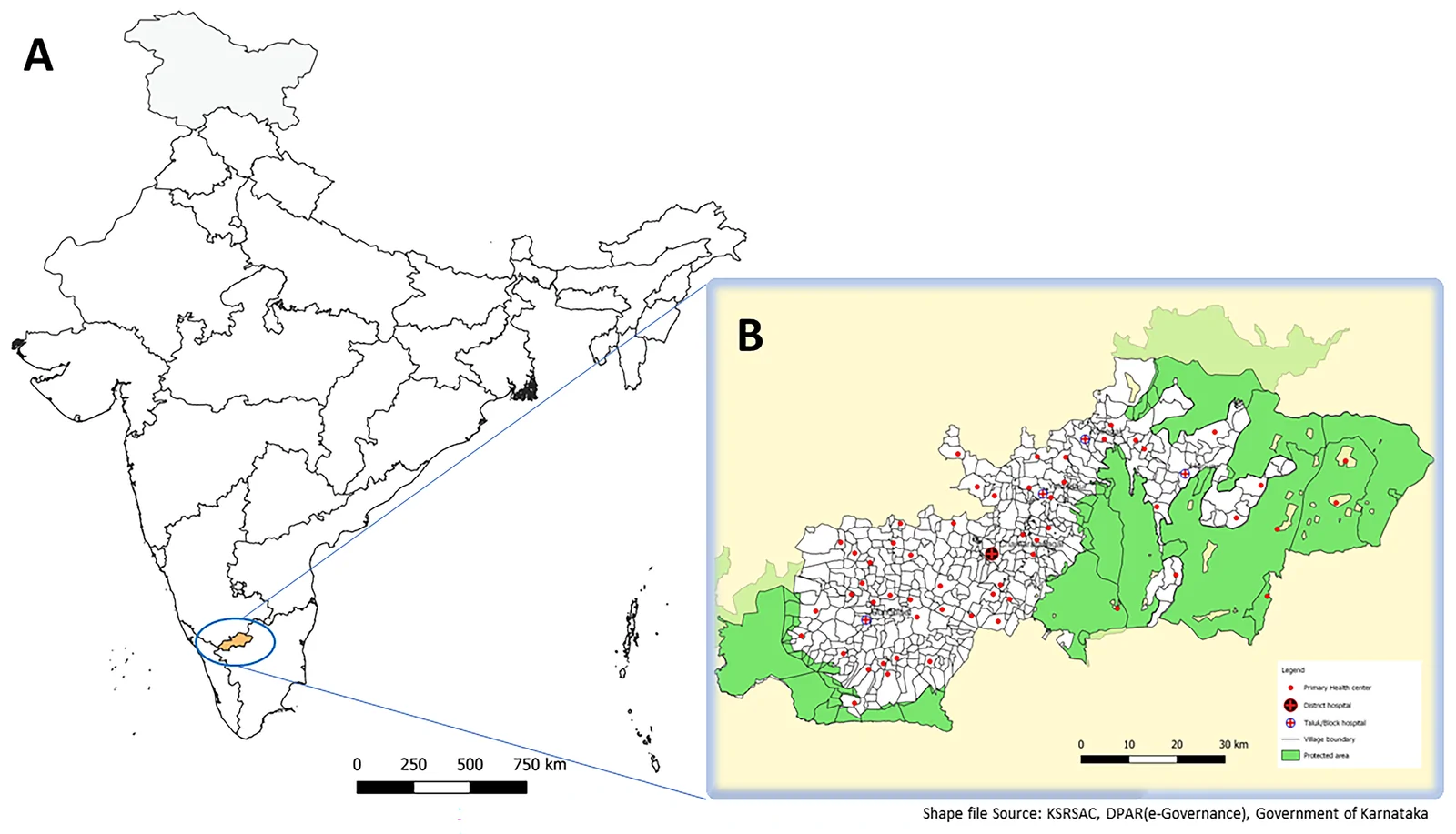
**A**) Chamarajanagar district study sites in Karnataka state, India **B**) Green colour indicates forest protected area, and the white area indicates the mainland of Chamarajanagar district.

**Study setting:** Chamarajanagar district is located in southern Karnataka, at the tri-junction of Karnataka, Tamil Nadu, and Kerala. Approximately 48% of the district's land is covered by protected forests, and 83% of its population (845,817 out of 1,098,473) resides in rural areas. The district is divided into five taluks or blocks: Hanur, Kollegal, Yelandur, Gundlupet, and Chamarajanagar ^34^. Primary healthcare in Chamarajanagar is provided by 60 primary health centres, three community health centres, three taluk or block hospitals, and one district hospital/medical college ( Figure 2). Local governance is administered through 126 Gram Panchayats. The educational landscape includes over 160 high schools and 66 pre-university colleges, spanning both public and private sectors. Law and order are maintained through 18 police stations.

### The Indian healthcare system's framework for Non-Communicable Diseases (NCDs)

India's healthcare system is characterised by a dualistic structure, comprising a multi-tiered public health apparatus and a substantial, parallel private sector ^35^. The public system is organised hierarchically to facilitate tiered healthcare delivery, with services guided by national health initiatives, such as the National Programme for Prevention and Control of Non-Communicable Diseases ^24, 36^. At the foundational primary level, AAM (Sub-Health Centres and Primary Health Centres) are increasingly strengthened under Ayushman Bharat—delivering Comprehensive Primary Health Care (CPHC) ^37^. These centres are responsible for a spectrum of services, including population enumeration, health education, follow-up, palliative care, and crucially, population-based screening for non-communicable diseases and common cancers as outlined by the NPNCD operational guidelines ^24, 36^.

Ascending the pyramid, the secondary and tertiary levels consist of District Hospitals and Medical Colleges. These institutions function as apex referral centres, providing advanced screening, definitive diagnosis, management of diseases and their complications, and specialised treatments ^38, 39^. The private sector mirrors this structure, with general practitioners and private clinics forming the primary point of contact and tertiary hospitals offering specialised care ^38^. Patient movement is fluid, occurring not only vertically within the public system but also horizontally between the public and private sectors. A significant characteristic of the overall system is its reliance on out-of-pocket expenditure, particularly for care sought in the private domain. While both public and private insurance mechanisms contribute to funding, especially at the tertiary level, a robust monitoring and evaluation framework is essential to oversee the performance and integration of this complex delivery system ^24^.

### Study design

We have used an implementation research design with a theory-driven inquiry to understand the complex phenomena ^40– 42^. These designs encompass multi-level analyses that examine the architecture and oversight of systems at the macro-level, the functioning of organisations and systemic interventions at the meso-level, and the roles of individuals involved in healthcare provision, utilisation, and governance at the micro-level. This multi-level approach acknowledges how these levels interact and shape each other, considering how decisions and behaviours at each level influence, and are influenced by the others. By examining causal mechanisms and context, these research designs offer a comprehensive understanding of complex interventions ^43^.

### Implementation plans

The IMPACT NCD project is implemented in collaboration with Chamarajanagar district Health and Family Welfare department, Chamarajanagar district Zilla Panchayath (District Development Council), Chamarajanagar District Police department, Department of Secondary Education, Department of Pre-University Education, and the tribal welfare department. The project is being executed in phases: implementation of objectives one and two occurred from January 2023 to July 2025, with objective three scheduled for the period from July 2025 to March 2026.

### Objective 1

***Improve health system capacity for the NCDs continuum of care.***

To improve the continuity of care for NCDs, the IMPACT NCD team focuses on Ayushman Arogya Mandir (primary health centre and sub-centres) activities, which include capacity building for healthcare personnel and introducing the concept of a continuum of care into primary healthcare at the Arogya Mandir.

Under the capacity building for health care workers like Accredited Social Health Activists (ASHAs), Primary Healthcare Officers (PHCOs), and Community Health Officers (CHOs), participatory workshops are conducted on prevention and screening (Community-Based Assessment Checklist activities) for diabetes, hypertension, and various cancers (oral, breast, and cervical).

At each Ayushman Arogya Mandir – Primary Health Centre, we will conduct participatory action workshops with CHOs (Community Health Officers) and medical officers on activities that support the continuum of care for hypertension and diabetes. These activities include 1) improving population-based screening and re-screening procedures in health facilities. 2) setting up a follow-up system that involves creating line lists of patients, issuing and maintaining follow-up booklets for patients, and integrating with the NCD portal for data management and continuum of care. Information, education, and communication (IEC) pamphlets (DOI: 10.6084/m9.figshare.23861616) and follow-up booklets (DOI: 10.6084/m9.figshare.23967393), developed under the project, are available in figshare ^44, 45^.

### Objective 2

We are conducting sensitisation sessions for Gram Panchayat (Village Council) elected members and staff under the Rural Development and Panchayat Raj Department (RDPR) and Adivasi leaders regarding the prevention of NCDs (Non-Communicable Diseases, including Diabetes, Hypertension, Cardiovascular diseases, Stroke, and COPD), health services, laws and regulations, and community mobilisation. For the Police department, we have been conducting training for all cadres on the Cigarettes and Other Tobacco Products (Prohibition of Advertisement and Regulation of Trade and Commerce, Production, Supply and Distribution) Act, 2003, and on alcohol regulation. We will utilise the learnings from the Comprehensive Tobacco Control Karnataka project, implemented by IPH Bengaluru. For secondary education students from 8 ^th^ standard to 10th standard and for Pre-University Education students from 11th and 12th grades, we are sensitising the students on the harmful use of tobacco and alcohol, the importance of healthy food, and physical activity as a part of primordial prevention. During the training, teachers and staff were also screened for hypertension and diabetes.

Objectives one and two were implemented from January 2023 to July 2025.

### Objective 3

Given the considerable scale of these multi-pronged interventions, particularly the scaling-up of strategies from previous research, it is essential to move beyond simply documenting outputs. To ensure effectiveness and sustainability in a challenging context like Chamarajanagar, this evaluation will critically examine the underlying pathways and mechanisms of change. It will investigate *how* the implemented continuum of care initiatives function in practice and *through what mechanisms* stakeholder sensitisation translates into tangible support for NCD prevention and control.

**Figure 3.**
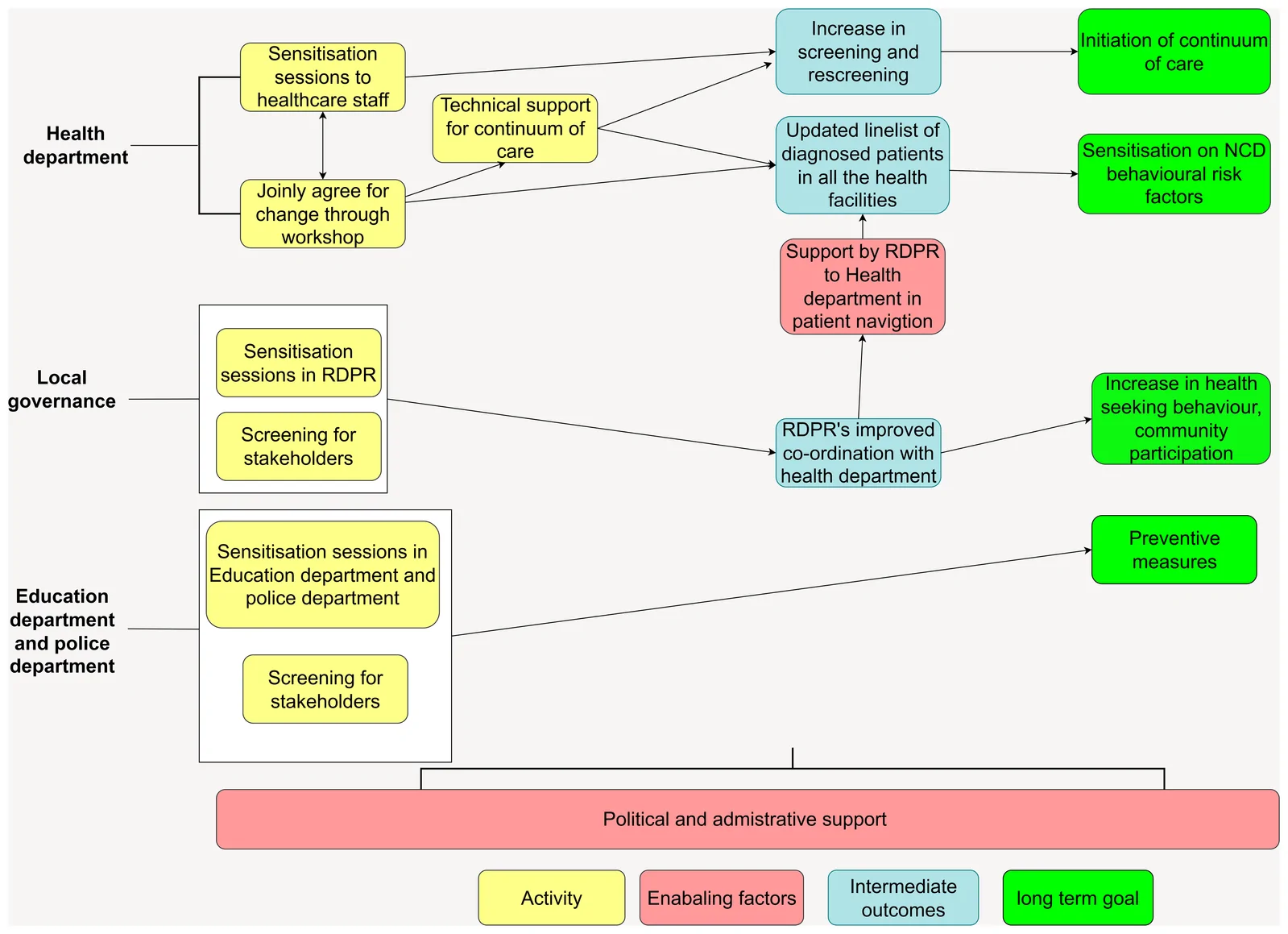
Initial Theory of Change.

This approach is crucial for determining who is most effectively reached under what specific conditions, which is vital for reducing NCD behavioural risk factors at the grassroots level. Such an analysis will provide vital insights for refining strategies, informing future multisectoral action, and ensuring that interventions effectively address the complex determinants of NCDs. To guide this inquiry, the IMPACT NCD project is designed as an implementation research study employing an iterative approach. The project commences with an initial program Theory of Change ( Figure 3), which posits hypotheses about the expected causal pathways from the interventions to the desired NCD prevention and control outcomes. These hypotheses will be systematically investigated and refined throughout the project's implementation and evaluation phases.

The study will employ a mixed-methods, embedded multiple-case study design, situated within an implementation research framework. This approach is designed to test and refine an *a priori* Theory of Change by providing a multi-level analysis of the intervention's implementation.

The initial stage involves a quantitative analysis of secondary program data encompassing key performance indicators such as NCD screening coverage, diagnosis rates, treatment follow-up, and other relevant engagement metrics ( Table 1). A sequential statistical approach will be employed. First, Principal Component Analysis (PCA) will be used to reduce the dimensionality of the variable set and identify latent constructs representing performance and context across all potential primary units. Subsequently, cluster analysis will be performed on the resultant principal components to empirically derive homogeneous strata of primary units based on their performance profiles.

**Table 1.** Key Performance Indicators.

Sl no	Facility	Comparable indicators to assess the performance
1.	Health Department - Ayushman Arogya Mandir	**NCD Portal Metrics (Quantifiable):**
		1. Percentage of people screened during the intervention
		2. Percentage of people followed up.
		3. Percentage of ABHA entries done during intervention
		4. The percentage of patients who started treatment during the intervention.
		**Descriptive/Contextual Factors for Assessment:**
		1. The difference between the total strength and the participants in the sensitisation session
		2. Infrastructure
		3. HR status in the facility
		4. Population of the area
		5. Transportation facilities
1.	High school and PU college	**Sensitisation Session Participation (Quantifiable):**
		1. Percentage of students who attended the sensitisation training
		**Descriptive/Contextual Factors for Assessment:**
		1. Transportation services to the facility
		2. Geographical aspect of the facility, indicating rural and urban
2.	Gram Panchayat	**Sensitisation Session Participation (Quantifiable):**
		1. Percentage of representatives who attended the sensitisation training
		**Descriptive/Contextual Factors for Assessment:**
		1. Transportation services to the facility
		2. Geographical aspect of the facility, indicating rural or urban
		3. Infrastructure
		4. HR status in the facility
3.	Police	**Sensitisation Session Participation (Quantifiable):**
		1. Percentage of staff who attended the sensitisation training
		**Descriptive/Contextual Factors for Assessment:**
		1. Transportation services to the facility
		2. Geographical aspect of the facility, indicating rural or urban
		3. Infrastructure
		4. HR status in the facility

From these empirically derived clusters, a purposive sampling strategy will be implemented to select nine to twelve primary units. This selection will be guided by the principle of maximum variation sampling to ensure the inclusion of cases that represent the full spectrum of implementation outcomes. The sample will comprise three to four units classified as high-performing (PHCs, GPs, Police, Schools) and three to four units classified as low-performing (PHCs, GPs, Police, Schools), based on the preceding quantitative analysis. All selected units will have received the NCD training and continuum of care support interventions.

**Figure 4.**
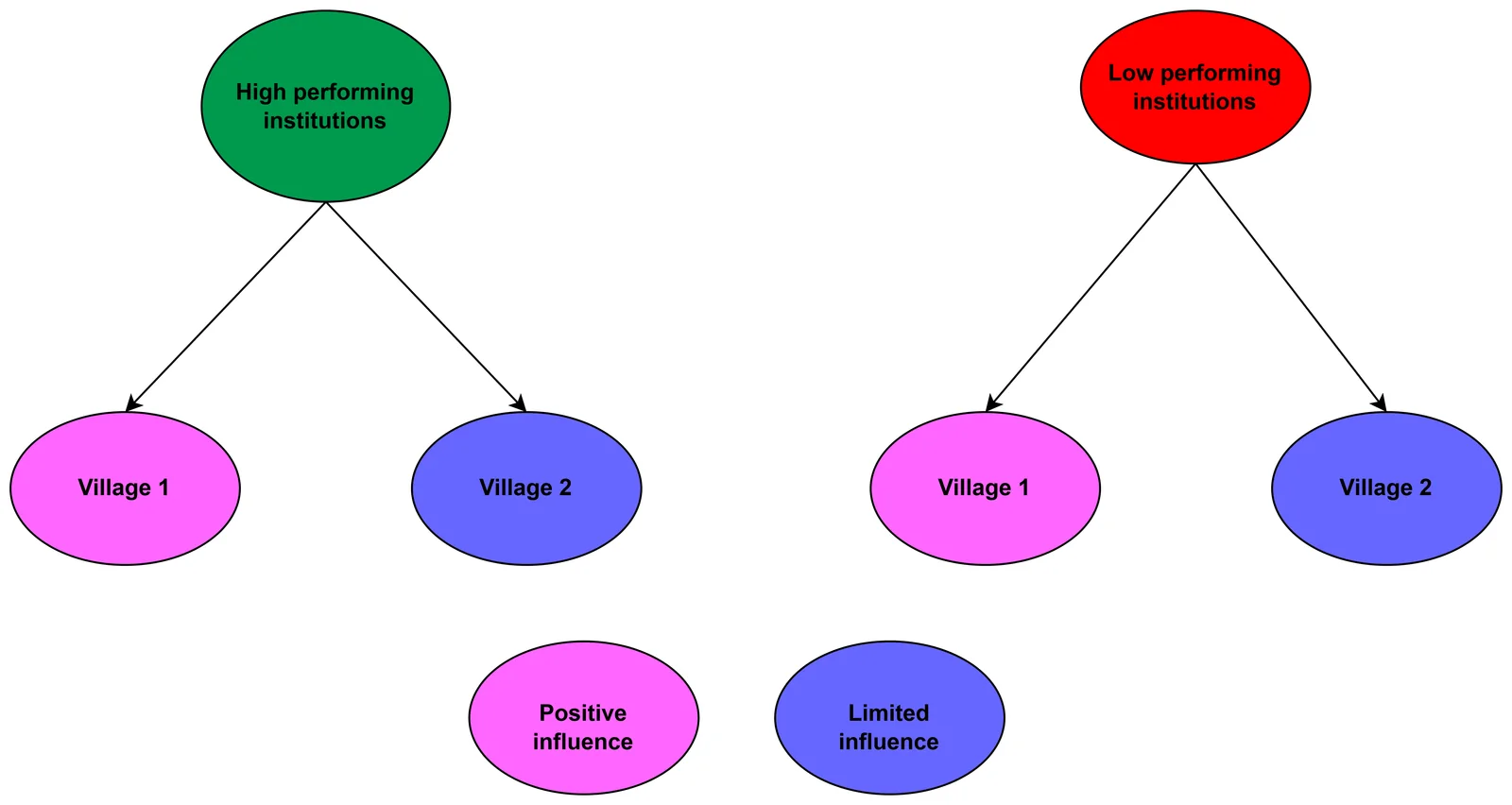
Case study selection technique.

The second stage of sampling involves the selection of embedded sub-units. Within each primary unit case, two villages will be purposively selected for in-depth qualitative inquiry ( Figure 4). The selection of these embedded cases will be based on prospectively defined criteria related to observable indicators of intervention effect. For instance, villages may be selected to represent exemplars of "positive influence" (e.g., high community engagement in NCD activities) and "limited influence" (e.g., minimal observable change), facilitating a comparative analysis of contextual facilitators and barriers.

This embedded, multiple-case study design enables a multi-level analysis. It facilitates a macro-level quantitative comparison across all units while simultaneously providing a micro-to-meso-level qualitative understanding of the causal mechanisms operating within specific contexts. This integrated approach is fundamental to the study's objective of rigorously testing and refining the a priori Theory of Change.

Qualitative Data Collection methods will be employed to elucidate the causal mechanisms, contextual factors, and processes that influence intervention outcomes.

- In-depth Interviews (IDIs): Semi-structured interviews will be conducted with key informants at the primary and sub-unit levels. These interviews will explore participants' experiences, perceptions, challenges, and facilitating factors related to the NCD continuum of care and multisectoral sensitisation efforts.
- Participant Observation (PO): Observations will be conducted within selected primary units, including Ayushman Arogya Mandirs, to understand the context, document the real-world implementation of NCD activities, and observe interactions between stakeholders and community members.

This design will allow for macro-level analysis using aggregated program secondary data across all units, while the embedded case studies will provide a micro-to-macro-level understanding by exploring the detailed pathways and mechanisms of change, or lack thereof, within specific contexts. Therefore, the overall evaluation will involve in-depth analysis of 9–12 primary unit cases and 18–24 embedded sub-unit analyses at the village level, utilising both quantitative and secondary qualitative data from the objective one and two methods to test and refine the project's theory of change.

## Procedures for data collection

To address the evaluation objective and understand the pathways and mechanisms of change as outlined in the theory of change, both quantitative and qualitative data will be collected from selected primary units and embedded sub-units.

**Quantitative Data Collection:** Quantitative program data will be collected from health centres and other relevant government departments. This data will primarily be sourced from existing records and program information systems. Quantitative data sources will include:

1. Data extracted from the NCD portal (e.g., numbers screened, diagnosed, on treatment, follow-up rates).
2. Data collected in objectives one and two as program data, which are captured at specific activities or for individual participants at the field level.
3. Relevant aggregate data from records maintained at PHCs, Gram Panchayats, Police Stations, and Schools about NCD-related activities, events (where available).

**Qualitative Data Collection:** Qualitative data will be collected to explore the "how" and "why" of observed changes, understand contextual factors, and gather insights into the process and mechanisms. Qualitative methods will include:

1. **In-depth Interviews:** key informants at the primary unit level, including health personnel (Medical Officers, Community Health Officers, PHCOs, ASHAs), Gram Panchayat members and officials, Police officers, school staff/teachers, and potential NCD patients and community members in the embedded villages. These interviews will explore experiences, perceptions, challenges, and facilitating factors related to the continuum of care, stakeholder sensitisation, and NCD prevention efforts.
2. **Participant Observation:** Carried out in selected primary units and in Ayush Arogya Mandirs to understand the context, observe interactions between stakeholders, and document the implementation of NCD-related activities and the functioning of the continuum of care in real-world settings.

All qualitative data from in-depth interviews and relevant observations will be audio-recorded (with informed consent). Transcriptions will be done in the local language and subsequently translated into English. The transcription and translation will be conducted by trained field staff/nurses, following a quality assurance process to ensure accuracy.

### Participant recruitment and procedures

Qualitative data collection will involve both in-depth interviews and participant observation. Participant recruitment and procedures will adhere to ethical guidelines, ensuring voluntary participation, informed consent, and confidentiality.

### In-depth interviews

**Participant Selection:** Interview participants will be purposively selected from the chosen primary units (clusters of institutions) and embedded villages to capture a range of perspectives relevant to the continuum of care and stakeholder sensitisation. Participants will include:

- Health personnel (Medical Officers, Community Health Officers (CHOs), Primary Health Care Officers (PHCOs), Accredited Social Health Activists (ASHAs)) involved in NCD care and follow-up.
- Key individuals from sensitised stakeholder groups within the primary units, such as Gram Panchayat elected members and officials, Police officers, and school staff/teachers who participated in sensitisation activities.
- Program beneficiaries, which will include individuals diagnosed with NCDs who are receiving care or follow-up, and potentially other community members in the embedded villages whose awareness or access to NCD prevention/care may have been influenced by the project.

**Recruitment and Consent Procedure:** Potential participants will be initially contacted via phone, through a designated contact person within their institution/village, or approached in person by the research team. The purpose of the study will be explained, and their willingness to participate will be assessed. If willing, a meeting will be scheduled at the participants' convenience and in a private setting to ensure confidentiality. Before conducting the interview, the research team will conduct a detailed informed consent process. This involves explaining the study's objectives, the purpose of the interview, how the data will be used, the procedures for ensuring confidentiality, the voluntary nature of participation, and their right to withdraw at any time without consequence. Written informed consent will be obtained from all participants; for those who are unable to write, thumbprint consent will be taken in the presence of a witness. If a participant declines to participate, their decision will be respected, and another eligible participant will be approached.

**Interview Process:** An in-depth interview guide will be used to explore participants' experiences, knowledge, perceptions, challenges, and suggestions related to the NCD continuum of care, stakeholder sensitisation activities, and their perceived impact on NCD prevention and control in their context. Interviews will be audio-recorded with the participant's explicit consent.

### Participant observation

**Observation Sites and Focus:** Participant observation will be conducted in selected primary units and embedded villages. The observation will focus on understanding the real-world implementation of the NCD continuum of care activities at health facilities and the nature of engagement and activities related to NCD prevention and sensitisation within Gram Panchayats, Police Stations, and Schools. The observer will also observe community dynamics and interactions related to NCDs in the embedded villages.

### Consent process in detail

Participants will receive an information sheet detailing the study's purpose, their selection process, and their right to withdraw at any time. For those who agree to participate, the interviewer will obtain written informed consent by asking them to sign a consent form. If a participant is illiterate, a researcher will read the document aloud. Oral consent will then be obtained and documented with the participant's thumbprint in the presence of a witness, who will also sign the consent form.

Participants will be informed that they will be free to withdraw from the study at any time with no impact on access to care, treatment (for patients), or their job (for health care providers). After the participant has had the opportunity to read through the informed consent form, he/she will be given an opportunity to ask further questions. The participant will then be asked open-ended questions about the information provided to ensure that he/she has understood the details of the study as well as the data management of the information sought during the interview.

The procedures concerning the study information and the informed consent will be performed by trained researchers who are not directly involved in the patient’s care or participation evaluation. This process will take place in a separate room whenever possible; it may take approximately 30 to 40 minutes.

### Data storage, security, backup, and archival

Training and other program activities photographs will be stored in a password-protected project computer at the IPH field station. Backup will be automatically done using a cloud-based backup service. Access to individual participant data is strictly limited to authorised research staff. To ensure participant confidentiality, personal identifying information, including names and participant identification numbers, will be omitted from all publications and conference presentations. IPH Bengaluru owns all the data.

### Data analysis strategy

The data analysis strategy is iterative and utilises a convergent mixed-methods design, guided by a theory-based evaluation approach to understand causal mechanisms.

 Theory of Change (ToC) as an Analytical Framework. The initial program ToC will be refined to serve as the central analytical framework. This refined ToC will map the hypothesised causal links between project interventions (Objectives 1 and 2) and a multi-level hierarchy of outcomes. Hypotheses derived from the ToC regarding how specific mechanisms operate will guide data collection and analysis.

### Qualitative data analysis

Qualitative data from in-depth interviews and field notes will be analysed using a systematic qualitative analytical approach, such as thematic analysis or framework analysis with ToC.

Analysis will involve several stages:

1. Data familiarisation: Reading and rereading transcripts and field notes to gain a deep understanding of the data.
2. Coding: Developing a coding framework, initially informed by the refined ToC and the research questions, and allowing for emergent themes. Codes will be applied to segments of text to categorise information related to implementation processes, experiences, perceptions, observed mechanisms, contextual influences, and outcomes.
3. Interpretation: Exploring relationships between codes and themes, identifying patterns and variations within and across different groups of participants (health staff, stakeholders, beneficiaries) and different types of primary units.
4. Cross-Case Analysis: Findings will be compared and contrasted across the different primary unit cases. This involves systematically examining similarities and differences in implementation processes, observed outcomes, the functioning of mechanisms, and the influence of contextual factors across the high-performing, low-performing, and baseline units. This comparative analysis will help identify patterns and draw broader conclusions about which pathways and mechanisms appear to be more effective, and under what conditions they are most likely to operate as intended. This step is crucial for addressing the " **under what conditions**" aspect of the objective.

### Ethics

Ethical approval for objectives one and two has been obtained from the Institutional Ethics Committee (IEC) of the Institute of Public Health, Bangalore (Study ID: IEC/I/FR/2022/13; Dated: 10 January 2023). A separate ethics submission for objective three was obtained on 18 August 2025 (Study ID: [IEC/I/FR/2022/13] 4/2025/ER).

Prior to enrolment, informed consent will be obtained from all participants, as detailed in the 'Consent Process' section.

## Discussion

The design of effective health interventions often navigates the tension between centralised strategies, which offer economies of scale, and decentralised approaches, which allow for crucial local adaptation ^45^. A hybrid model that leverages the strengths of both can yield robust and sustainable outcomes.

This project is strategically positioned at the confluence of these models. We are leveraging a centralised repository of knowledge and best practices derived from IPH Bengaluru's extensive fieldwork on the THETA, INCARE, and Comprehensive Tobacco Control projects. This evidence base is then operationalised through a deeply collaborative, decentralised partnership with the Chamarajanagar district authorities. This implementation modality is intended not merely to achieve project objectives but to build lasting institutional capacity by embedding proven strategies directly into the local health system, thereby enhancing the potential for long-term sustainability and scalable impact.

## Data Availability

No data is associated with this article.
