# Supplementary material for: Strengthening the continuum of care and multisectoral action for non-communicable diseases in Chamarajanagar district, India: an implementation research protocol (IMPACT NCD)
Source: Wellcome Open Res. 2025 Oct 29;10:609. [Version 1] doi: 10.12688/wellcomeopenres.24926.1 (PMC13560808; doi:10.12688/wellcomeopenres.24926.1)
Supplement: Peer review history

# Reviewer report: Reviewer response for version 1

**DOI:** 10.21956/wellcomeopenres.27456.r140690

**Published:** 2025-12-30

**Authors:** Kibachio Joseph Mwangi

**Affiliations:** 1 Division of Non-Communicable Diseases, Ministry of Health, Nairobi, Kenya

This is a study protocol for the IMPACT NCD project in Chamarajanagar district, Karnataka, India, aiming to strengthen the primary care continuum of care for hypertension/diabetes and multisectoral action via sensitisation/training of local governance, police, and students.

 The protocol emphasises implementation evaluation to understand “what works, for whom, and why,” using a theory-driven approach with a Theory of Change (ToC).

**1) Rationale for, and objectives of, the study clearly described?: Partly**

- This protocol clearly motivates the problem (rising NCD burden, health-system gaps) and links the work to prior district research, including specific system gaps (enumeration form issues, inadequate follow-up, substandard screening/diagnosis).
- The broad project aim and implementation-evaluation intent are well articulated.
- The objectives are described at a high level, the protocol needs to clearly articulate specific evaluation questions and especially specifying primary vs secondary implementation outcomes.
- May need to add a section listing the primary and secondary implementation outcomes and how each maps to ToC as well as articulate how causality/cause and effect will be articulated and judged.

**2) Is the study design appropriate for the research question: Yes**

- Well-articulated but please clarify as the protocol describes sequential mixed methods but later describes a convergent mixed-methods design.
- I suggest you use a sequential explanatory mixed method which is a two-phase design starting with broad quantitative data (numbers, surveys) to find patterns, then using targeted qualitative data (interviews, focus groups) to explain why those patterns occurred, answering "what happened?" first, then "why/how?"

**3) Are sufficient details of the methods provided to allow replication by others? Partly**

- Please indicate the rules you will use for handling of missing data/outliers.
- To improve the validity of the  qualitative analysis approach please pre-specify the analytic approach with inductive codes, coding reliability procedures eg double-coding proportion and the methods of reconciliation.
- Provide explicit selection criteria and a rubric for classifying “high/low performance” and “positive/limited influence to sharpen the sampling technique.
- Add a sentence clarifying whether any qualitative participants will be <18; if yes, detail assent/guardian consent and safeguarding procedures.

Is the study design appropriate for the research question?

Yes

Is the rationale for, and objectives of, the study clearly described?

Partly

Are sufficient details of the methods provided to allow replication by others?

Partly

Are the datasets clearly presented in a useable and accessible format?

Not applicable

Reviewer Expertise:

Global Health, Public health response as well as policy imperatives for Chronic Diseases especially in resource constrained contexts.

I confirm that I have read this submission and believe that I have an appropriate level of expertise to confirm that it is of an acceptable scientific standard.

# Response

**Published:** 2026-06-21

**Authors:** Yogish Channa Basappa

**Affiliations:** Health system strengthening, Institute of Public Health Bengaluru, Bengaluru, Karnataka, India

We thank Dr Kibachio Joseph Mwangi for the careful and constructive assessment. The comments have materially strengthened the protocol. This includes the articulation of the evaluation questions and outcomes, the specification of the quantitative stratification method, and the level of detail provided for replication. We revised the manuscript accordingly and respond to each point below. Section names refer to the revised version.

**1. The objectives are described at a high level; please articulate specific evaluation questions and specify primary versus secondary implementation outcomes, with mapping to the Theory of Change and a statement of how causality will be judged.**

 We added a new section titled "Evaluation question and implementation assessment". It states the specific evaluation questions regarding how health system capacity building combined with technical and hands-on support translates into sustained patient follow-up, and how stakeholder sensitisation influences local awareness and engagement. It distinguishes the primary implementation dimensions of adoption and fidelity from the secondary dimensions of acceptability and feasibility. Primary dimensions are assessed through routine programme data with proxy programme indicators and qualitative exploration of how staff integrate the tools into daily workflows. Secondary dimensions are captured through qualitative exploration of lived experience. The section makes explicit that causal inference rests on a generative logic rather than experimental attribution. We examine how the introduction of project resources triggers specific behavioural or systemic mechanisms within a given context to produce the observed outcomes, documenting context-mechanism-outcome configurations across high-performing and low-performing units identified through programme and project data.

**2. The design is described as sequential mixed methods in one place and convergent in another; please clarify and consider a sequential explanatory design.**

 We thank the reviewer for identifying this inconsistency, which we corrected. The study is described as a sequential explanatory mixed-methods design. A quantitative phase stratifies primary units by performance applying Latent Profile Analysis and a qualitative phase explains the observed patterns. We removed the earlier reference to a "convergent" design in the Data analysis strategy section. A new "Mixed-methods Integration Strategy" subsection clarifies that integration occurs at two points. The quantitative stratification frames the qualitative sampling. At the interpretive stage, we will apply a triangulation approach to merge these datasets through a joint display matrix that maps programme metrics against the emergent qualitative themes for each high-performing and low-performing unit.

**3a. Please indicate the rules for handling missing data and outliers.**

 We added explicit rules to the Statistical Estimation and Stratification subsection. Facility records with incomplete core indicators are handled applying complete-case analysis for the profile model. The number and characteristics of any excluded units are reported. Outlying standardized values are checked against the source records and are retained, corrected, or excluded only where they reflect data-entry error.

**3b. Please pre-specify the analytic approach with inductive codes and coding reliability procedures, including the double-coding proportion and the method of reconciliation.**

 We specified these procedures in the Qualitative data analysis section. The analysis is framework analysis anchored in the refined Theory of Change, combining deductive codes derived from the framework with inductive codes for emergent themes.

**3c. Please provide explicit selection criteria and a rubric for classifying high and low performance and positive and limited influence.**

 Performance classification is specified objectively in the Statistical Estimation and Stratification subsection through Latent Profile Analysis. The optimal performance model is selected applying the Bayesian Information Criterion and the Bootstrap Likelihood Ratio Test. Units are assigned to their modal profile applying posterior class-membership probabilities at a 0.70 threshold generated by this statistical model. High-performing and low-performing units are those with modal membership in the profiles showing the highest and lowest mean values on the standardised performance indicators. At the embedded village level, "positive influence" and "limited influence" cases are distinguished applying prospectively defined community-responsiveness criteria. These criteria contrast villages with high engagement in NCD activities against those with minimal participation, as set out in the Qualitative case selection section.

**3d. Please clarify whether any qualitative participants will be under 18; if so, detail assent, guardian consent, and safeguarding.**

 We confirm all participants in the qualitative evaluation are adults including health personnel, Gram Panchayat members and officials, police personnel, schoolteachers and administrative staff, and adult programme beneficiaries. Students take part only in the Objective 2 sensitization activities and are not enrolled as research participants in the evaluation. The school-side interviewees are teachers and staff. No participant in the qualitative evaluation is under 18. Assent or guardian consent for research participation does not arise. We believe these revisions address the substantive concerns raised and have improved the clarity, rigour, and replicability of the protocol. We thank the reviewer again for the time and expertise.

# Reviewer report: Reviewer response for version 1

**DOI:** 10.21956/wellcomeopenres.27456.r138163

**Published:** 2025-12-18

**Authors:** Kanica Kaushal

**Affiliations:** 1 Institute of Liver and Biliary Sciences, New Delhi, India

This protocol describes the IMPACT NCD project, an implementation research study in Chamarajanagar district, Karnataka, India, aiming to strengthen primary healthcare capacity for NCD prevention and management. The study has three objectives: improving health system capacity for NCD continuum of care, sensitizing multisectoral stakeholders, and conducting implementation evaluation using a theory-driven mixed-methods design.

 Strengths

- Comprehensive background situating the work within India's NCD burden and policy context
- Builds logically on prior research (INCARE, THETA projects)
- Appropriate theory-driven implementation research design
- Sound sequential mixed-methods approach combining PCA/cluster analysis with qualitative case studies
- Ethical approvals obtained and materials made publicly available via figshare

Points Requiring Revision

**Rationale/Objectives (Partly):**

- Objectives need greater specificity as measurable research questions
- The knowledge gap addressed beyond previous studies should be more explicitly stated

**Methods (Partly):**

- Sample size justification missing for 9-12 primary units and 18-24 sub-units
- PCA/cluster analysis specifications incomplete (variables, clustering criteria, performance thresholds)
- Number of planned interviews per stakeholder category not specified
- Interview guides and observation protocols not provided
- Specific hypotheses derived from Theory of Change not articulated
- Mixed-methods integration strategy not described
- Intervention content and format insufficiently detailed for replication

Recommendation

**Revisions required.** Authors should provide sample size justification, elaborate quantitative analysis specifications, specify qualitative sampling, articulate testable hypotheses, and describe mixed-methods integration procedures.

Is the study design appropriate for the research question?

Yes

Is the rationale for, and objectives of, the study clearly described?

Partly

Are sufficient details of the methods provided to allow replication by others?

Partly

Are the datasets clearly presented in a useable and accessible format?

Not applicable

Reviewer Expertise:

Liver health and Implementation research

I confirm that I have read this submission and believe that I have an appropriate level of expertise to confirm that it is of an acceptable scientific standard, however I have significant reservations, as outlined above.

# Response

**Published:** 2026-06-21

**Authors:** Yogish Channa Basappa

**Affiliations:** Health system strengthening, Institute of Public Health Bengaluru, Bengaluru, Karnataka, India

We thank Dr Kanica Kaushal for the constructive assessment. The comments have strengthened the protocol. We revised the manuscript accordingly and address each point below.

**Rationale and objectives — objectives need greater specificity as measurable research questions, and the knowledge gap beyond previous studies should be stated more explicitly.**

 Response: We agree with this observation. The new "Evaluation question and implementation assessment" section translates the objectives as specific evaluation questions with defined primary and secondary implementation dimensions. The Theory of Change section now articulates the testable hypotheses. The Introduction states the knowledge gap explicitly by noting that global literature on multisectoral action originates predominantly from high-income countries and overlooks the socioeconomic realities of rural India. IMPACT NCD addresses this by investigating causal pathways to generate contextual evidence on integrating multisectoral strategies within government systems.

**Methods — sample size justification missing for the 9–12 primary units and 18–24 sub-units.**

 Response: The Qualitative case selection section now justifies the sample based on the principle of information power rather than thematic saturation. We select four to six high-performing and four to six low-performing primary health facilities to capture the full spectrum of implementation outcomes and achieve theoretical replication. We select two villages within the catchment of each selected primary health facility to yield eighteen to twenty-four villages. This provides a volume sufficient to capture local contextual variation.

**Methods — PCA and cluster analysis specifications incomplete (variables, clustering criteria, performance thresholds).**

 Response: We shifted from Principal Component Analysis to Latent Profile Analysis to provide a model-based classification better suited for identifying distinct performance strata. We added the full specification to the manuscript. Input variables includes clinical and administrative indicators including individuals aged 30 and above enrolled, screened, diagnosed, initiated on treatment, and linked to Ayushman Bharat Health Accounts. These are combined with implementation metrics like follow-up booklets distributed and line-list creation alongside standardised contextual factors includes density covariates for ASHA, Community Health Officer. The "Statistical Estimation and Stratification" subsection details the specific estimation parameters, model selection criteria, and performance thresholds.

**Methods — number of planned interviews per stakeholder category not specified.**

 Response: The Participant Selection section now states a target of 35 to 45 in-depth interviews to maximise representation across categories. The breakdown includes nine to twelve Medical Officers or Community Health Officers, nine to twelve ASHAs or Primary Health Care Officers, nine to twelve programme beneficiaries, and three to four each among Gram Panchayat officials or elected members, police personnel, and school teachers or administrative staff. These are drawn equally from the high-performing and low-performing strata.

**Methods — interview guides and observation protocols not provided.**

 Response: We have uploaded the full in-depth interview guide and the observation checklist as extended data on figshare, where the project pamphlet and follow-up booklet are already available.

**Methods — mixed-methods integration strategy not described.**

 Response: A new "Mixed-methods Integration Strategy" subsection describes integration at the sampling stage and at the interpretive stage is explained in protocol.

**Methods — intervention content and format insufficiently detailed for replication.**

 Response: The intervention descriptions are expanded. Objective 1 specifies structured half-day participatory workshops with a curriculum covering national screening guidelines, standardisation of blood pressure measurement, and hands-on data-entry training delivered through didactic presentation and role-play. Objective 2 states session durations including three to four hours for Gram Panchayat members and staff and two to three hours for all police cadres. It identifies the enforcement focus under the Cigarettes and Other Tobacco Products Act 2003 and broader non-communicable disease prevention. The core messaging and presentation formats are standardised across the district. Given the participatory nature of these workshops, a detailed analysis of their implementation dynamics will be provided in a subsequent manuscript.

 We trust these revisions comprehensively address the points raised
